# Incidence of circumcision among insured adults in the United States

**DOI:** 10.1371/journal.pone.0275207

**Published:** 2022-10-17

**Authors:** Behnam Nabavizadeh, Kevin D. Li, Nizar Hakam, Nathan M. Shaw, Michael S. Leapman, Benjamin N. Breyer

**Affiliations:** 1 Department of Urology, Weill Cornell Medicine, New York, New York, United States of America; 2 Department of Urology, University of California San Francisco, San Francisco, California, United States of America; 3 Department of Urology, Yale University School of Medicine, New Haven, Connecticut, United States of America; 4 Department of Biostatistics and Epidemiology, University of California San Francisco, San Francisco, California, United States of America; PLOS: Public Library of Science, UNITED KINGDOM

## Abstract

**Purpose:**

Although circumcision is the most commonly performed surgery in males, less is known about the incidence and indications of adult circumcision. In this study, we aim to present the incidence of adult circumcision across the United States.

**Methods:**

Using IBM MarketScan^®^ Commercial Database from 2015 to 2018, we obtained claims for circumcision in men between 18 and 64 years of age. We calculated the incidence of adult circumcision over the study period and across the United States. We also collected data on indications for surgery using International Classification of Diseases codes.

**Results:**

We identified a total of 12,298 claims for adult circumcisions. The mean age was 39 (±12.9) years. The average incidence rates remained relatively constant from 98.1 per 100,000 person-years in 2015 to 98.2 per 100,000 person-years in 2018 (Δ+0.1%). The age-standardized incidence rates varied significantly across the United States (from 0 to 194.8 per 100,000 person-years) with South Dakota having the highest rate. The most common indications for adult circumcision were phimosis (52.5%), routine/ritual circumcision (28.7%), phimosis + balanitis/balanoposthitis (6.8%), balanitis (3.8%) and balanoposthitis (2.6%), and significantly varied by age groups.

**Conclusion:**

This study suggested a wide geographic variation in rates of adult circumcision between states with highest incidences in the Northeast United States. Future studies can identify the underlying causes for the observed variations.

## 1. Introduction

Circumcision, the surgical removal of some or all of the foreskin from the penis, is the most commonly performed surgical procedure worldwide [1]. In the United States (US), it is estimated that up to 80% of men are circumcised [2]. The procedure has been associated with multiple health benefits including reduced risks of urinary tract infections, HIV/sexually transmitted infections (STIs), and penile cancer, particularly in the context of neonatal circumcision [3]. As such, despite persisting arguments about its health benefits and potential harms on sexual function, multiple professional societies such as the American Academy of Pediatrics and American Urological Association have recently concluded that the health benefits of neonatal circumcision outweigh risks and that the procedure is generally safe [3,4].

Comparatively little data exists for adult circumcision relative to studies on neonatal circumcision. Most adult circumcision is done electively for a number of surgical indications, including phimosis, balanitis, condyloma, or dyspareunia [5]. Patients may also elect to undergo adult circumcision due to religious, cosmetic, or social motivations as well as disease prevention [6]. While randomized control trials have shown the benefits of adult circumcision in reducing incidence of HIV and STIs, generalizability of these trials to settings with low rates of HIV and STIs such as the US is limited [7–9]. Meanwhile, multiple survey studies have highlighted potential adverse effects on sexual functioning after adult circumcision [10–13].

Epidemiologic data is needed to better inform research on indications, causal relationships, motivating factors, risks, and benefits of circumcision in adults. While recent reports have characterized trends in neonatal circumcision [14], there are no national studies that offer comprehensive data about incidence and characteristics of adult circumcision in the US. We aim to present incidence data on the practice of adult circumcision across the country in men with commercial insurance. We hypothesize significant age and geographic variations exist in incidence of adult circumcision across the US.

## 2. Subject and methods

### 2.1 Database

We obtained data from IBM MarketScan^®^ Commercial Database which contains health insurance claims across the continuum of care (e.g. inpatient, outpatient, etc.) as well as enrollment data from large employers and health plans across the US [15]. This database contains over 245 million unique patients since 1995 and can be used to create a nationally representative data sample of Americans with employer-provided health insurance [15]. It also records demographic, diagnostic, and procedural data. MarketScan data are deidentified, therefore this study was deemed exempt from obtaining institutional review board approval.

### 2.2 Study population

We used International Classification of Diseases (ICD) ninth (V50.2) and tenth (Z41.2) revisions and Current Procedural Terminology (CPT; 54150, 54160, 54161) codes to identify adult males between 18 and 64 years of age who had undergone circumcision in the US between January 1, 2015 and December 31, 2018. We also used ICD codes for diagnosis variables as follows to identify indications for circumcision: phimosis (ICD9: 605; ICD10: N47.1), routine/ritual circumcision (ICD9: V50.2; ICD10: Z41.2), balanitis (ICD9: 607.81; ICD10: N48.1), balanoposthitis (ICD9: 607.1; ICD10: N47.6).

### 2.3 Data analysis

We used descriptive statistics to characterize the study cohort. We reported continuous variables as means and standard deviations (SD) and categorical variables were reported as frequencies and percentages. We calculated the average incidence rate of adult circumcision using the mid-year numbers of insured males between 18 and 64 years of age enrolled in the MarketScan database. In order to calculate the population at risk for the denominator (i.e., uncircumcised males ≥18 years), we obtained the rate of neonatal circumcision in different US regions for the years before 2000 [16]. Then, we multiplied the mid-year numbers of insured males and [1-neonatal circumcision rate] to calculate the population at risk. Moreover, we used data from the US Census Bureau to perform the direct method of age adjustment for incidences [17]. We reported incidences as number of circumcisions per 100,000 person-years. Chi-square test was used to test the association between diagnoses and age groups or geographic regions. STATA 14.2 was used to perform all statistical tests. All *p* values were two-sided and considered to be statistically significant if <0.05. The STROBE (Strengthening the Reporting of Observational Studies in Epidemiology) statement was followed for the design and reporting of this study [18].

## 3. Results

**Fig 1:**
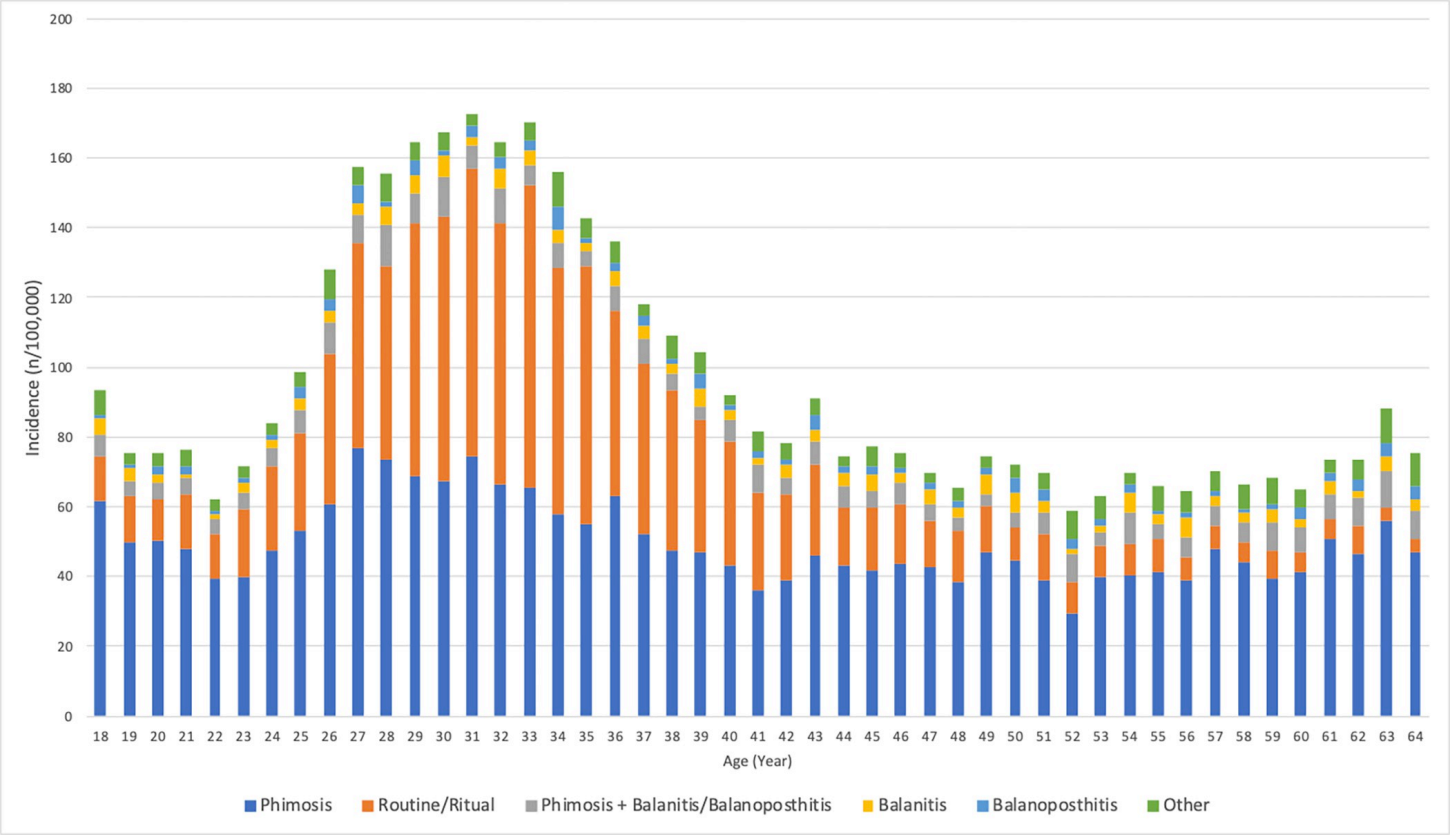
Incidence of adult circumcision across the age range defined by indications.

We identified a total of 12,298 claims for circumcision cases. The mean age of patients undergoing circumcision was 39 (±12.9) years. S1 Fig demonstrates the age distribution of the patients. More than a third of the patients (34.1%) were born between the years 1980 and 1990. The average incidence rates remained relatively constant from 98.1 per 100,000 in 2015 to 98.2 per 100,000 in 2018 (Δ+0.1%; Table 1). The indications for adult circumcision were phimosis (52.5%), routine/ritual circumcision (28.7%), phimosis + balanitis/balanoposthitis (6.8%), balanitis (3.8%), and balanoposthitis (2.6%). Fig 1 represents the incidence of adult circumcision across the age range defined by diagnoses. Indications for circumcision had significantly different distributions across the age groups (*p*<0.01; Table 2). While phimosis constituted 49.1% of the indications for adult circumcision between ages 18 to 34, 63.8% of patients aged 55 to 64 had such a diagnosis.

**Table 1:** Characteristics of study participants.

**Total number of patients**	12,298
**Total average incidence rate, n/100,000 person-years**	99.7
**Age, mean (SD)**	39.0 (12.9)
**Year of surgery, n (%)** **2015** **2016** **2017** **2018**	3,337 (27.1) 3,267 (26.6) 2,832 (23.0) 2,862 (23.3)
**Average incidence rates, n/100,000 person-years** **2015** **2016** **2017** **2018**	98.1 98.5 104.7 98.2
**Indications, n (%)** **Phimosis** **Routine/Ritual** **Phimosis + Balanitis/Balanoposthitis** **Balanitis** **Balanoposthitis** **Other**	6,453 (52.5) 3,523 (28.7) 839 (6.8) 463 (3.8) 321 (2.6) 699 (5.7)
**Geographic region, n (%); Age-standardized incidence rates, (n/100,000 person-years)** **Northeast** **North Central** **South** **West** **Unknown**	2,513 (20.4); 125.1 1,730 (14.1); 110.1 6,052 (49.2); 101.0 1,944 (15.8); 61.0 59 (0.5); N/A
**Type of service, n (%)** **Inpatient** **Outpatient**	272 (2.2) 12,026 (97.8)
**Type of insurance plan, n (%)** **Comprehensive** **EPO** **HMO** **POS** **PPO** **POS with capitation** **CDHP** **HDHP** **Unknown**	246 (2.0) 106 (0.9) 1,389 (11.3) 941 (7.7) 6,653 (54.1) 237 (1.9) 1,330 (10.8) 1,079 (8.8) 317 (2.6)

CDHP, consumer driven health plan; EPO, exclusive provider organization; HDHP, high deductible health plan; HMO, health maintenance organization; POS, point of service; PPO, preferred provider organization; SD, standard deviation.

**Table 2:** Percentage of each indication for adult circumcision stratified by the age groups (*p*<0.01).

	Phimosis	Routine/Ritual	Phimosis + Balanitis/Balanoposthitis	Balanitis	Balanoposthitis	Other
**18–34**	49.1	35.7	5.8	3.0	2.2	4.3
**35–44**	46.0	37.9	5.7	3.3	2.3	4.8
**45–54**	58.5	18.0	7.9	5.5	3.6	6.6
**55–64**	63.8	9.0	9.9	4.7	3.1	9.5

Values may not add up to 100 due to rounding errors.

**Fig 2:**
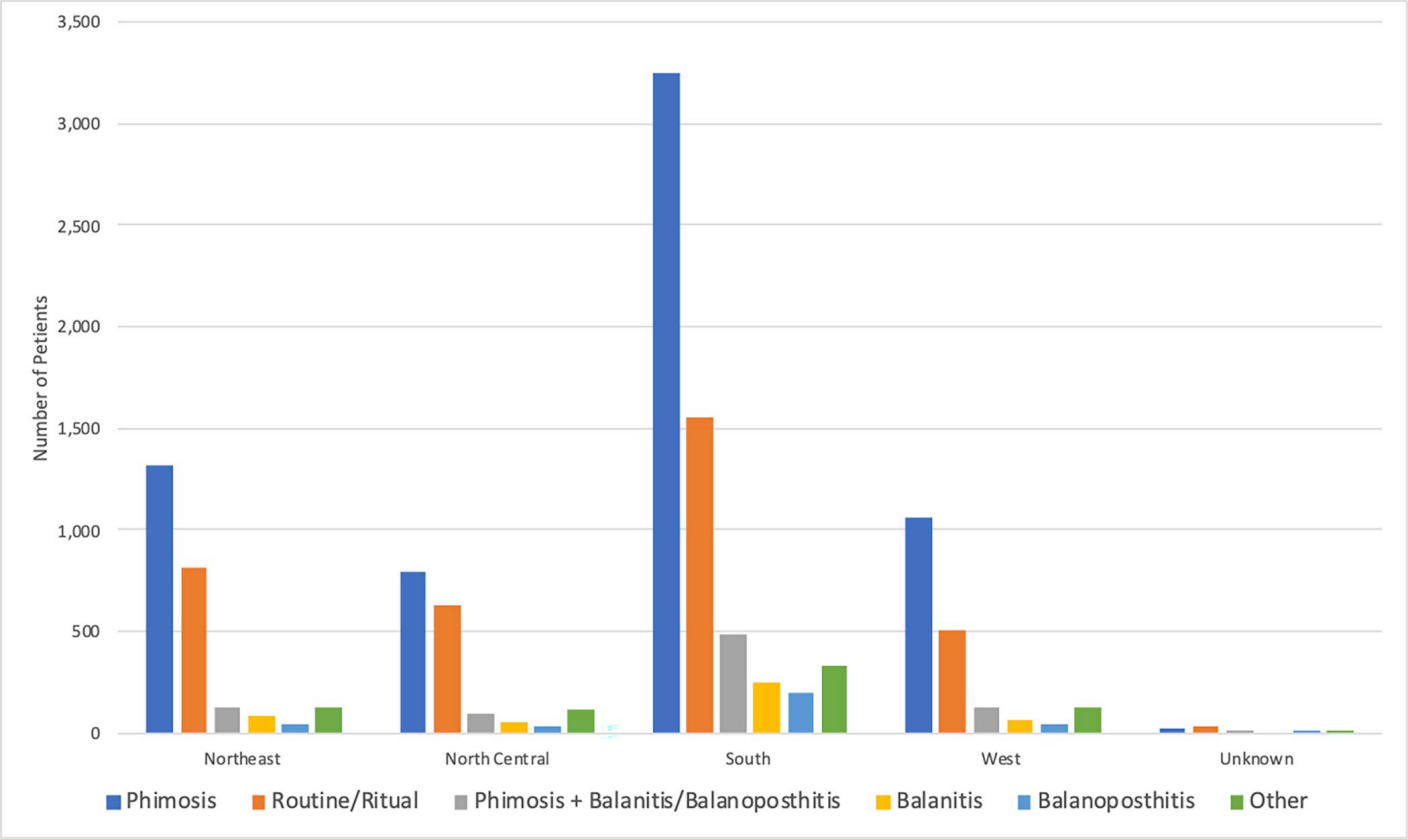
Indications for adult circumcision stratified by the United States regions (*p*<0.01).

Almost a half of surgeries (49.2%) were done in the South region while 20.4%, 15.8%, and 14.1% were performed in the Northeast, West, and North Central regions, respectively. However, the age-standardized incidence rate was highest in Northeast (125.1 per 100,000), followed by North Central (110.1 per 100,000), South (101.0 per 100,000), and West (61.0 per 100,000). In addition, indications were significantly different across the US regions (*p*<0.01; Fig 2). Almost all surgeries were done in outpatient settings (97.8%; Table 1). Preferred provider organization (PPO) 6,653 (54.1%) was the most common insurance plan used for adult circumcision in the US.

**Fig 3:**
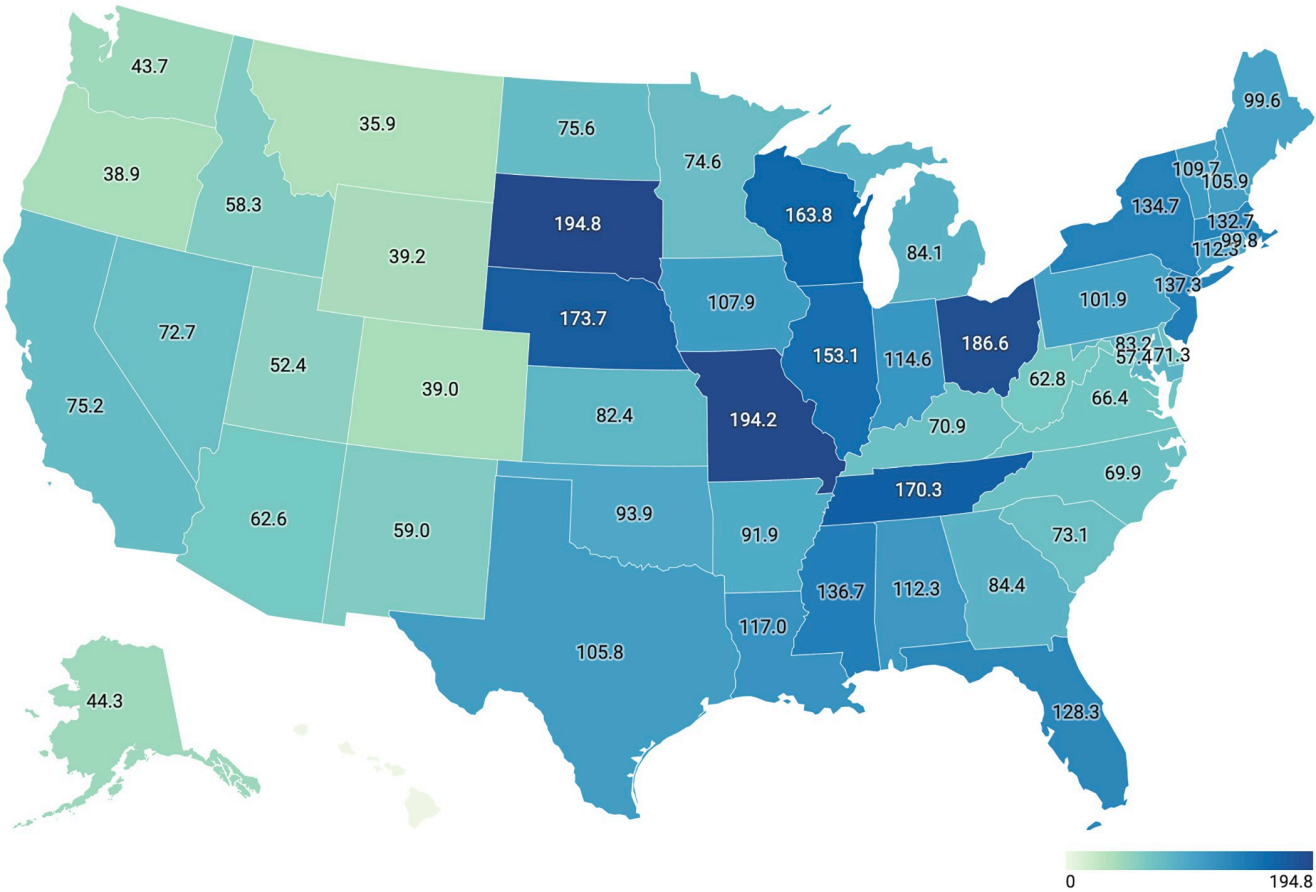
Choropleth map of the United States showing age-standardized incidence rates of adult circumcision in each state.

The age-standardized incidence rates of adult circumcision stratified by states is displayed in Fig 3. The top 5 States include South Dakota (194.8/100,000), Missouri (194.2/100,000), Ohio (186.6/100,000), Nebraska (173.7/100,000), and Tennessee (170.3/100,000). In contrast, Hawaii (0/100,000; no cases of circumcision in adult males were recorded in the MarketScan database during the study period. Of note, only 3,964 beneficiaries from Hawaii were found in the dataset during the study period), Montana (35.9/100,000), Oregon (38.9/100,000), Colorado (39.0/100,000), and Wyoming (39.2/100,000) had the lowest age-standardized incidence rates.

## 4. Discussion

To our knowledge, this is the first study that reports the incidence and characteristics of adult circumcision in the US. Overall, circumcision is a relatively uncommon surgery among American adult men with a total average incidence rate of 99.7 per 100,000 person-years. This may reflect the high prevalence of neonatal circumcision in the US [19]. In this sample of adult circumcisions, patients had a mean age of 39 years, were most often circumcised for phimosis, and commonly resided in Southern geographic states. The age-standardized incidence rates of circumcision varied from 0 to 194.8 per 100,000 person-years between US states with South Dakota having the highest rate.

We found that most adult circumcisions were performed for phimosis. This was consistent with other published studies that described indications for adult circumcision, showing that phimosis was the indication for circumcision in roughly half of cases. Similarly, balanitis was a common indication, responsible for 14.4–17% of adult circumcisions in published studies [5,11]. Notably, we found that 28.7% of adult circumcisions in our study were performed for routine or ritual indications. This potentially indicates that while adult circumcision may be performed for its health benefits in regions with high endemicity of HIV and other STIs, it may be more commonly performed for religious or aesthetic purposes in the US.

Diagnoses associated with adult circumcision varied significantly by age group. Phimosis represented the largest proportion of diagnoses in patients aged 18–34 and its role as an indication for adult circumcision increased with older age groups. However, the average incidence rates of adult circumcision as a result of phimosis decreased with the increasing age of the patients (Fig 1). This may reflect the natural history of the condition which may resolve spontaneously with age or be treated with circumcision if not resolved by early adulthood, or it may portend the fact that the patient grows accustomed to the issue [20]. A similar trend was observed for routine/ritual circumcisions, which made up a smaller proportion of cases in older age groups. This may be explained by patients opting for circumcision in early adulthood if they were previously uncircumcised as minors. This may also be the results of societal norms requiring males to be circumcised by a given age. Alternatively, the increase in rates of circumcision may coincide with greater sexual activity and subsequent sexual issues such as dyspareunia, a common indication for circumcision [5].

Another contributor to the observed heterogeneity in the age distribution of the patients could be the fact that male circumcision was not uniformly practiced at the time of their birth. Following physician’s advocacy for the benefits of circumcision in early 20^th^ century, it became a routine surgery in the US [21]. However, in 1971 the American Academy of Pediatrics Task Force recommended against the routine neonatal circumcision stating that circumcision “offered no medical benefit during the neonatal period” [22]. Subsequent studies showed that the prevalence of circumcision had decreased significantly among the American men born in 1980s (84%) compared to those born in 1970s (91%) [23]. Interestingly, in our study more than a third of patients were born in 1980s which can imply a lower prevalence of neonatal circumcision among this age demographic.

Our study had several limitations that should be acknowledged. As an insurance claim database, MarketScan does not report several demographics and clinical variables including race. As a result, we were not able to assess the racial disparities in adult circumcision. Additionally, as a claims database diagnoses like “phimosis” rely on coding from a heterogenous provider base and may not represent a similarly diverse clinical severity that is not captured. Lastly, our study investigated adult males with commercial insurances. MarketScan does not include 1/3 of population with public insurances. In addition, about 8% of US population are uninsured who were not included in our analysis [24]. Therefore, our results might not be generalized to the whole US population. Despite these limitations, this study provided novel insights on adult circumcision in the US at a national scale.

## 5. Conclusions

The adult circumcision is relatively an uncommon surgery among American adult men in certain states. Phimosis remained the most common indication for circumcision in adults. Indications varied significantly across the age ranges and geographic regions. There is a wide variation in age-standardized incidence rates of circumcision across the US, with South Dakota, Missouri, and Ohio having the highest rates. The Northeast region had the highest incidence of circumcision in adult men. Future epidemiologic studies can investigate the possible reasons for the observed regional variations in this study.

## Supporting information

S1 Fig Age distribution of the study participants. (PNG)

## References

[1] Perera CL, Bridgewater FH, Thavaneswaran P, Maddern GJ. Safety and efficacy of nontherapeutic male circumcision: a systematic review. Ann Fam Med. 2010;8(1):64–72. Epub 2010/01/13. doi: 10.1370/afm.1073 ; PubMed Central PMCID: PMC2807391. 20065281 PMC2807391

[2] Morris BJ, Wamai RG, Henebeng EB, Tobian AA, Klausner JD, Banerjee J, et al. Estimation of country-specific and global prevalence of male circumcision. Popul Health Metr. 2016;14:4. Epub 2016/03/05. doi: 10.1186/s12963-016-0073-5 ; PubMed Central PMCID: PMC4772313. 26933388 PMC4772313

[3] Male circumcision. Pediatrics. 2012;130(3):e756–85. Epub 2012/08/29. doi: 10.1542/peds.2012-1990 . 22926175

[4] Circumcision—American Urological Association. https://www.auanet.org/guidelines/guidelines/circumcision Accessed August 15, 2021.

[5] Siev M, Keheila M, Motamedinia P, Smith A. Indications for adult circumcision: a contemporary analysis. Can J Urol. 2016;23(2):8204–8. Epub 2016/04/18. . 27085824

[6] Collier R. Late cuts: an international look at adult circumcision. Cmaj. 2012;184(1):E15–6. Epub 2011/11/23. doi: 10.1503/cmaj.109-4013 ; PubMed Central PMCID: PMC3255211. 22105758 PMC3255211

[7] Auvert B, Taljaard D, Lagarde E, Sobngwi-Tambekou J, Sitta R, Puren A. Randomized, controlled intervention trial of male circumcision for reduction of HIV infection risk: the ANRS 1265 Trial. PLoS Med. 2005;2(11):e298. Epub 2005/10/20. doi: 10.1371/journal.pmed.0020298 ; PubMed Central PMCID: PMC1262556. 16231970 PMC1262556

[8] Gray RH, Kigozi G, Serwadda D, Makumbi F, Watya S, Nalugoda F, et al. Male circumcision for HIV prevention in men in Rakai, Uganda: a randomised trial. Lancet. 2007;369(9562):657–66. Epub 2007/02/27. doi: 10.1016/S0140-6736(07)60313-4 . 17321311

[9] Friedman B, Khoury J, Petersiel N, Yahalomi T, Paul M, Neuberger A. Pros and cons of circumcision: an evidence-based overview. Clin Microbiol Infect. 2016;22(9):768–74. Epub 2016/08/09. doi: 10.1016/j.cmi.2016.07.030 . 27497811

[10] Dias J, Freitas R, Amorim R, Espiridião P, Xambre L, Ferraz L. Adult circumcision and male sexual health: a retrospective analysis. Andrologia. 2014;46(5):459–64. Epub 2013/04/23. doi: 10.1111/and.12101 . 23600924

[11] Fink KS, Carson CC, DeVellis RF. Adult circumcision outcomes study: effect on erectile function, penile sensitivity, sexual activity and satisfaction. J Urol. 2002;167(5):2113–6. Epub 2002/04/17. . 11956453

[12] Rai BP, Qureshi A, Kadi N, Donat R. How painful is adult circumcision? A prospective, observational cohort study. J Urol. 2013;189(6):2237–42. Epub 2013/01/02. doi: 10.1016/j.juro.2012.12.062 . 23276514

[13] Kim D, Pang MG. The effect of male circumcision on sexuality. BJU Int. 2007;99(3):619–22. Epub 2006/12/13. doi: 10.1111/j.1464-410X.2006.06646.x . 17155977

[14] Jacobson DL, Balmert LC, Holl JL, Rosoklija I, Davis MM, Johnson EK. Nationwide Circumcision Trends: 2003 to 2016. J Urol. 2021;205(1):257–63. Epub 2020/07/28. doi: 10.1097/JU.0000000000001316 . 32716676

[15] IBM MarketScan Research Databases for Health Services Researchers (White Paper), IBM Watson Health®. https://www.ibm.com/downloads/cas/6KNYVVQ2 Accessed August 15, 2021.

[16] Owings M, Uddin S, Williams S. Trends in circumcision for male newborns in US hospitals: 1979–2010. National Center for Health Statistics. 2013.

[17] US Census Bureau. nc-est2020-agesex-res: Annual Estimates of the Resident Population by Single Year of Age and. Sex for the United States: April 1, 2010 to July 1, 2020.

[18] Vandenbroucke JP, von Elm E, Altman DG, Gøtzsche PC, Mulrow CD, Pocock SJ, et al. Strengthening the Reporting of Observational Studies in Epidemiology (STROBE): explanation and elaboration. Epidemiology. 2007;18(6):805–35. Epub 2007/12/01. doi: 10.1097/EDE.0b013e3181577511 . 18049195

[19] Introcaso CE, Xu F, Kilmarx PH, Zaidi A, Markowitz LE. Prevalence of circumcision among men and boys aged 14 to 59 years in the United States, National Health and Nutrition Examination Surveys 2005–2010. Sex Transm Dis. 2013;40(7):521–5. Epub 2013/08/24. doi: 10.1097/01.OLQ.0000430797.56499.0d . 23965763

[20] Morris BJ, Matthews JG, Krieger JN. Prevalence of Phimosis in Males of All Ages: Systematic Review. Urology. 2020;135:124–32. Epub 2019/10/28. doi: 10.1016/j.urology.2019.10.003 . 31655079

[21] Alanis MC, Lucidi RS. Neonatal circumcision: a review of the world’s oldest and most controversial operation. Obstet Gynecol Surv. 2004;59(5):379–95. Epub 2004/04/21. doi: 10.1097/00006254-200405000-00026 . 15097799

[22] Fetus AAoPCo Newborn. Standards and recommendations for hospital care of newborn infants: American Academy of Pediatrics; 1971.

[23] Xu F, Markowitz LE, Sternberg MR, Aral SO. Prevalence of circumcision and herpes simplex virus type 2 infection in men in the United States: the National Health and Nutrition Examination Survey (NHANES), 1999–2004. Sex Transm Dis. 2007;34(7):479–84. Epub 2007/04/07. doi: 10.1097/01.olq.0000253335.41841.04 . 17413536

[24] Berchick ER, Hood E, Barnett JC. Health insurance coverage in the United States: 2018: Washington, DC: US Department of Commerce; 2019.

