# Supplementary figures and images for: Incidence of circumcision among insured adults in the United States
Source: PLoS One. 2022 Oct 17;17(10):e0275207. doi: 10.1371/journal.pone.0275207 (PMC9576047; doi:10.1371/journal.pone.0275207)

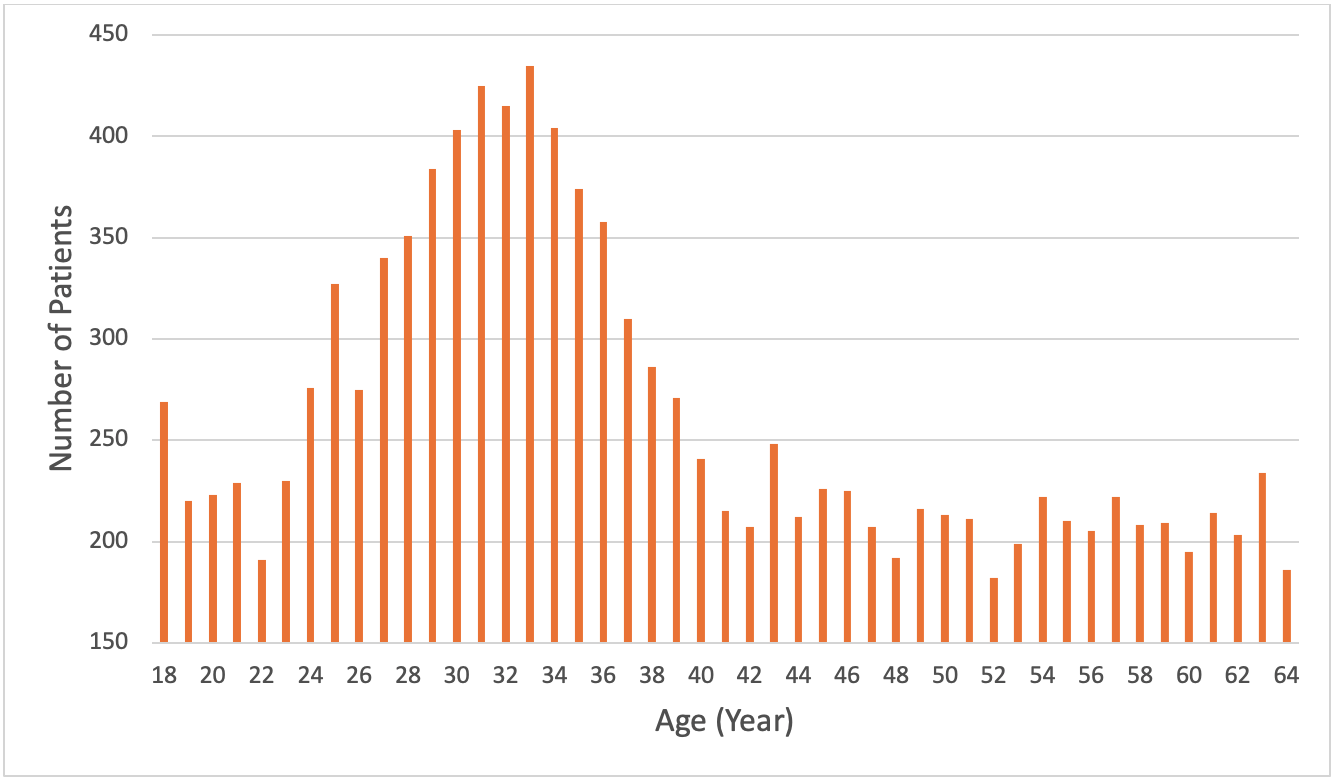

Supplement: S1 Fig — (PNG) [file pone.0275207.s001.png]
